# Extracellular vesicle signalling in perioperative neurocognitive disorders

**DOI:** 10.3389/fimmu.2026.1849953

**Published:** 2026-06-02

**Authors:** Jian Li, Min Yu

**Affiliations:** 1 Department of General Surgery, Lanzhou University Second Hospital, Lanzhou University, Lanzhou, Gansu, China; 2 Department of Anesthesiology, Lanzhou University Second Hospital, Lanzhou University, Lanzhou, Gansu, China

**Keywords:** BBB, erioperative neurocognitive disorders, extracellular vesicle, neuroinflamamation, PND

## Abstract

Perioperative neurocognitive disorders (PND), including postoperative delirium, delayed neurocognitive recovery, and postoperative neurocognitive disorder, are important complications in older and vulnerable surgical patients. These conditions are associated with prolonged hospitalization, reduced functional recovery, greater healthcare utilization, and worse longer-term outcomes. Current evidence indicates that PND is not driven by a single mechanism. Instead, systemic inflammation, neurovascular dysfunction, blood-brain barrier injury, glial activation, innate immune signalling, and synaptic injury are all thought to contribute. However, the pathways by which peripheral perioperative stress is translated into sustained postoperative brain dysfunction remain incompletely understood. Extracellular vesicles (EVs) have attracted increasing attention in this context. As lipid bilayer-enclosed particles carrying proteins, lipids, and nucleic acids, EVs are involved in intercellular and inter-organ communication and may provide a mechanistic link between surgical injury and downstream cerebral responses. Emerging evidence suggests that EV-associated signals may participate in the progression from peripheral inflammation and vascular stress to blood-brain barrier dysfunction, neuroinflammation, complement-related synaptic injury, and neuronal dysfunction. In parallel, EV-associated cargo may offer a biologically informative peripheral signal for perioperative studies. This review summarizes the biological basis of EV signalling, major methodological issues relevant to EV research, and current clinical and experimental evidence linking EV-associated signals to PND. It also discusses source-specific EV populations and their potential relevance to perioperative brain injury.

## 1 Introduction

Perioperative neurocognitive disorders (PND) encompass postoperative delirium, delayed neurocognitive recovery, and postoperative neurocognitive disorder (1). Clinically, these syndromes matter because they cluster in older and vulnerable surgical patients and are associated with prolonged hospitalization, functional decline, greater healthcare use, and worse longer-term outcomes (2). Mechanistic work no longer supports a single-cause model. Instead, current evidence points to interacting processes that include systemic inflammation, blood–brain barrier injury, glial activation, neurotransmitter dysregulation, complement-related synaptic injury, and age-related cerebral vulnerability (3–6). What remains less well defined is how a predominantly systemic perioperative insult is converted into sustained and spatially organized signalling within the brain.

Extracellular vesicles (EVs) provide a plausible framework for this missing link. This does not imply that EVs replace established peripheral-to-brain signalling mechanisms, such as soluble cytokines, damage-associated molecular patterns, neuroendocrine and autonomic pathways, or blood–brain barrier dysfunction. Rather, EVs may add a distinct signalling layer to these processes. EVs are membrane-bound particles released by virtually all cell types and can carry proteins, lipids, and nucleic acids that reflect the physiological or pathological state of the donor cell (7). Their significance lies not only in cargo diversity, but also in their ability to protect labile molecules in body fluids, reach distant tissues, and modify the phenotype of recipient cells (7). Compared with freely soluble mediators, EVs can package multiple classes of biological information within the same vesicle population and may partially preserve information about the activation state and possible cellular origin of the releasing cell. In neurological disease, EVs are increasingly regarded as mediators of brain–periphery communication rather than passive by-products of cell injury (8). This perspective is particularly relevant to PND, because it offers a mechanistic way to connect peripheral tissue injury, vascular-interface dysfunction, innate immune activation, and secondary neural injury within a single signalling model.

Evidence specific to the perioperative setting is now beginning to accumulate. Clinical studies have linked perioperative EV cargo, including EV-associated microRNAs, proteins, metabolites, and complement-related signals, with postoperative delirium or poor postoperative neurocognitive outcomes (9–11). Experimental studies further suggest that postoperative or cell-derived EVs can influence neuroinflammation and cognition in animal models (12, 13). Together with a recent British Journal of Anaesthesia editorial emphasizing the relevance of EV signalling in postoperative neurocognitive dysfunction (14), these findings support EVs as an emerging research direction in PND. This review discusses the role of EV signalling in PND, with emphasis on its biological basis, relationship with established peripheral-to-brain signalling mechanisms, biomarker value, contribution to disease mechanisms, and translational prospects.

## 2 Clinical phenotype and perioperative context of PND

Perioperative neurocognitive disorders are clinically heterogeneous rather than a single postoperative brain syndrome. According to current nomenclature, this spectrum includes postoperative delirium, delayed neurocognitive recovery, and postoperative neurocognitive disorder, which differ in clinical presentation, assessment window, and likely underlying biology (1, 3, 15). Postoperative delirium is typically an early, acute, and fluctuating syndrome, whereas delayed neurocognitive recovery and postoperative neurocognitive disorder refer to more sustained decline detected over later postoperative intervals (3). This distinction is important in both clinical and research settings, because cognitive changes identified before surgery, during the immediate postoperative period, or weeks to months later do not necessarily represent the same biological process (15). The perioperative setting adds a further layer of heterogeneity. Older age, frailty, baseline cognitive vulnerability, vascular comorbidity, and pre-existing low-grade systemic inflammation have all been associated with increased susceptibility to postoperative delirium or broader PND phenotypes (16, 17). In parallel, the biological burden imposed by surgery is not uniform. Cardiac surgery, major orthopaedic procedures, spine surgery, and other major operations differ in the degree of tissue injury, haemodynamic perturbation, transfusion exposure, and inflammatory activation, all of which may influence the timing, magnitude, and interpretation of postoperative circulating signals (15). For this reason, findings in this field are best interpreted within a phenotype-defined, time-aware, and procedure-specific framework rather than generalized across all perioperative cognitive outcomes.

Current studies on PND have focused mainly on two broad categories of circulating signals. One comprises inflammatory mediators, including C-reactive protein (CRP), IL-6, TNF-α, and related cytokine networks, which are commonly used to reflect the systemic inflammatory response associated with surgery and delirium risk (18). The other includes markers linked to neural, glial, or neurovascular injury, such as neurofilament light, glial fibrillary acidic protein (GFAP), and indices of blood-brain barrier dysfunction, which may better reflect perioperative brain vulnerability and ongoing tissue stress (19–21). Although these approaches have advanced understanding of PND, most soluble markers capture only one aspect of a complex perioperative response and are strongly influenced by sampling time, surgical magnitude, and baseline comorbidity. In this context, extracellular vesicles are of particular interest, because they may provide a more integrated signal and help explain how systemic perioperative stress is translated into neurovascular injury, glial activation, and neuronal dysfunction.

## 3 Biological basis of extracellular vesicle signalling

### 3.1 Biology of extracellular vesicles

Extracellular vesicles (EVs) is now the preferred generic term for cell-derived particles enclosed by a lipid bilayer and lacking replicative capacity (7). This terminology is important because, in most experimental settings, isolated vesicles cannot be assigned with certainty to a single biogenetic pathway (22). In broad terms, small EVs are commonly associated with the endosomal system, whereas larger vesicles are more often generated by outward budding of the plasma membrane (23). EV release is not a random by-product of membrane turnover. Rather, it is influenced by cell type, cell state, and local conditions such as inflammation, hypoxia, metabolic stress, and tissue injury (24). Another central feature of EV biology is that their cargo is selectively organized rather than passively inherited from the donor cell. EVs can contain proteins, lipids, metabolites, DNA, mRNA, microRNAs, and other non-coding RNAs, but their composition does not simply mirror the intracellular environment (24). Cargo incorporation is shaped by sorting machinery, membrane domains, RNA-binding proteins, and the physiological state of the releasing cell, which helps explain why EV populations differ across tissues and pathological contexts (25). This selective packaging is one of the reasons EVs are considered functionally relevant signalling particles rather than inert cellular debris.

After release, EVs can influence recipient cells through several routes. Some act through ligand–receptor interactions at the cell surface, whereas others are internalized by endocytosis, phagocytosis, macropinocytosis, or, in some settings, direct membrane fusion (26). Their biological effects depend not only on cellular uptake, but also on intracellular trafficking and whether functional cargo is successfully released inside the recipient cell (27). In the central nervous system, EVs participate in communication among neurons, glial cells, and vascular cells, and they are increasingly implicated in signalling between the brain and the periphery (8, 28). However, although EVs can interact with the blood-brain barrier and may cross it under some conditions, direct evidence for consistent bidirectional transport *in vivo* remains limited, and the underlying mechanisms appear to vary across models and inflammatory states (8, 9). In the context of PND, the key issue is therefore not simply the presence of circulating EVs, but whether perioperative changes in EV release and cargo are sufficient to influence neurovascular, glial, or neuronal function in a biologically meaningful way.

### 3.2 Methodological considerations in extracellular vesicle studies

A first challenge in EV research is that classification itself is partly operational rather than absolute. Current guidelines recommend using extracellular vesicles as the generic term and describing vesicles by measurable features such as size, density, molecular composition, or cell of origin whenever biogenesis cannot be demonstrated directly (7). In practice, investigators often distinguish small EVs from medium/large EVs and may refer to exosomes, microvesicles, or apoptotic bodies, but these labels are not interchangeable and are not always justified by the experimental data (22). Small EVs are often enriched for vesicles of endosomal origin, whereas larger vesicles more commonly arise through plasma membrane budding; however, post-isolation samples usually contain heterogeneous and overlapping populations rather than a single, pure subtype (22). This point has direct interpretative consequences: differences reported between studies may reflect enrichment of different EV fractions rather than true biological disagreement. For this reason, both MISEV2023 and the recent Nature Reviews Methods Primers article stress that EV preparations should be described with methodological precision, rather than by assuming a specific vesicle identity from terminology alone (22). A second issue concerns separation and characterization. No single method is optimal for all biofluids or downstream applications. Ultracentrifugation, size-exclusion chromatography, density gradients, polymer-based precipitation, affinity capture, and microfluidic platforms differ substantially in yield, purity, scalability, and subtype bias (22). As a result, particle counts, protein concentration, or the detection of a few conventional markers are insufficient on their own to establish EV identity or sample quality (22). These limitations are especially important in blood-derived EV studies, which are highly relevant to PND. Blood contains abundant lipoproteins, protein complexes, residual platelets, and cell fragments that overlap with EVs in size and composition, making complete purification unrealistic (29, 30). The MIBlood-EV statement therefore highlights pre-analytical variables—such as anticoagulant choice, time to processing, centrifugation steps, storage conditions, and freeze-thaw cycles—as major determinants of data quality (29, 30). One particularly important confounder is ex vivo platelet activation. Comparative studies have shown that serum-derived EV fractions contain more particles and are enriched in platelet-associated proteins relative to plasma-derived fractions, indicating that coagulation itself can reshape the measured vesicle pool (30). Likewise, lipoprotein contamination can distort proteomic and functional analyses, and recent work has shown that density-based lipoprotein depletion can substantially improve plasma EV readouts (31). In perioperative studies, where coagulation, haemodilution, inflammation, transfusion, and tissue injury are all dynamic, these technical variables are likely to be even more influential. Interpretative problems extend beyond isolation. Bulk circulating EV preparations rarely allow confident assignment of tissue or cellular origin, and many workflows enrich only a subset of the vesicle spectrum (32). Consequently, a change in EV cargo does not necessarily indicate a true change in a defined biological population. Similar caution is needed in functional studies. Uptake assays may be affected by dye artifacts, aggregation, or nonspecific adsorption, and evidence of vesicle internalization does not by itself prove biologically meaningful cargo transfer (7). Current recommendations therefore emphasize orthogonal characterization, appropriate negative and process controls, and, where possible, perturbation experiments that distinguish EV-dependent effects from those caused by co-isolated non-vesicular material (7). Transparent reporting is also essential. EV-TRACK was established to improve methodological transparency and reproducibility across the field, and its rationale remains highly relevant for translational work (33). In the context of PND, where clinical studies are still limited in number and often involve small cohorts, technical and interpretative discipline is particularly important; otherwise, pre-analytical noise and compositional ambiguity may be mistaken for mechanistic insight.

## 4 Extracellular vesicles as biomarkers in PND

### 4.1 Circulating extracellular vesicles and perioperative risk stratification

Risk stratification is the most immediate clinical entry point for EV-based biomarkers in PND. In current practice, perioperative cognitive risk is still estimated mainly from demographic and clinical features, including advanced age, frailty, pre-existing cognitive impairment, comorbidity burden, and surgical stress (1). These variables remain useful, but they do not directly capture the biological susceptibility of the brain to perioperative injury. Recent biomarker-focused reviews have therefore emphasized the need for molecular tools that can complement conventional clinical assessment and help identify vulnerable patients before overt postoperative cognitive manifestations appear (17, 18, 34). This need is reinforced by observational data showing that preoperative frailty and cognitive impairment are associated with postoperative delirium, yet neither performs well as a stand-alone predictor, and broader integrated models remain necessary (35). Likewise, preoperative low-grade systemic inflammation has been linked to subsequent postoperative delirium (POD) and postoperative cognitive decline in older adults, suggesting that biological vulnerability is already present before surgery and may be measurable in peripheral samples (16). Against this background, circulating EVs are attractive because they are readily accessible in blood, can integrate signals from multiple tissues, and may enrich low-abundance molecular information that is diluted or unstable in whole plasma or serum. EV-based markers should not be viewed as simple replacements for conventional inflammatory markers such as CRP, IL-6, or TNF-α. Their potential advantage is not necessarily earlier detection or uniform brain specificity, but the ability to provide complementary molecular information through multi-cargo content, relative cargo stability, partial cellular source information, and links to several PND-related processes, including inflammation, neurovascular injury, glial activation, and synaptic stress.

Direct evidence for EV-based perioperative risk stratification remains limited but is beginning to take shape. In a matched cohort of older adults undergoing spine surgery, Cho et al. analyzed pre-event plasma EV microRNAs and identified 142 differentially expressed miRNAs in patients who later developed postoperative delirium; the top 10 candidates, including miR-548ar-5p and miR-627-5p, were already elevated before delirium onset and were subsequently tested in an independent validation set (9). This study is important not because it establishes a ready-to-use assay, but because it shows that circulating EV cargo may reflect latent perioperative susceptibility rather than merely postoperative injury. A subsequent prospective study extended this concept to bacterial extracellular vesicles (BEVs) in preoperative blood samples from elderly patients undergoing spinal surgery (36). In that cohort, a random-forest classifier built from significant circulating BEV taxa showed the lowest out-of-bag error rate compared with models based on baseline laboratory variables or gut microbiome profiles, and external validation yielded an accuracy of 80%; Moraxellaceae- and Acinetobacter-associated EVs emerged as the most informative taxa (36). Taken together, these studies suggest that circulating EV signals may contribute to biological risk enrichment before clinical deterioration becomes evident. At the same time, the evidence remains narrow, largely single-center, and concentrated in older spine-surgery populations. At present, circulating EVs are better viewed as candidate adjuncts to multimodal perioperative risk assessment than as replacement biomarkers for clinical decision-making.

### 4.2 Dynamic changes in extracellular vesicle cargo after surgery and anaesthesia

A key difference from preoperative risk markers is that postoperative EV signals are dynamic rather than fixed. Experimental studies indicate that surgery and anaesthesia can remodel circulating EV cargo in a time-dependent manner. In a mouse orthopaedic trauma model, serum EV content changed at 6, 24, and 72 h after surgery, with altered miRNA and protein profiles linked to extracellular matrix remodelling and metabolic pathways (37). More recent work in aged mice showed that circulating EVs after anaesthesia and surgery carried distinct proteins and miRNAs and were biologically active in recipient animals (12). In a related aged-rat model, plasma exosomal miR-182-5p was reported to promote neuroinflammation and cognitive dysfunction through BDNF suppression and NF-κB activation, further supporting the functional relevance of postoperative EV remodelling (38).

Human studies also suggest that perioperative EV cargo changes over time, although the available evidence remains limited. In patients undergoing hip or knee replacement, serial plasma and cerebrospinal fluid sampling showed that poor postoperative neurocognitive outcome was associated with changes in EV cargo, including miRNAs and complement-related proteins, particularly EV-associated C3; these findings support the value of longitudinal sampling but do not fully resolve cellular source attribution (11). In cardiac surgery, plasma exosome multi-omics identified postoperative protein and metabolite alterations, including changes in MMP9, TLR2, ICAM1, S100B, and pathways related to neuroinflammation and blood–brain barrier dysfunction in patients with postoperative delirium; these signals should be interpreted in the context of a complex perioperative milieu that includes coagulation activation, haemodilution, inflammatory responses, and mixed circulating vesicle populations (10). In geriatric hip-fracture patients, perioperative changes in plasma exosomal α-synuclein correlated with delirium severity and IL-6 changes, suggesting that EV-associated signals may reflect clinical intensity rather than merely case status, although interpretation remains limited by unresolved issues related to vesicle purity, cellular source, and perioperative inflammatory confounding (39). Taken together, these studies suggest that surgery and anaesthesia induce phase-specific EV cargo remodelling, and that serial perioperative sampling is likely to provide more information than a single postoperative measurement.

### 4.3 Diagnostic and prognostic potential of extracellular vesicle-associated signals

For EV-associated signals to be clinically useful in PND, they must do more than reflect perioperative stress; they should help distinguish patients with relevant neurocognitive injury and, ideally, indicate likely clinical trajectory. This is the main rationale for considering EVs as diagnostic or prognostic biomarkers rather than simply mechanistic correlates. Recent biomarker reviews in POD/PND have emphasized that clinically meaningful markers should be accessible from peripheral biofluids, allow repeated sampling, and map onto biologically relevant processes such as neuronal injury, neuroinflammation, endothelial dysfunction, and blood–brain barrier disruption (40). EVs are attractive in this regard because they concentrate molecular cargo that may be difficult to detect in unfractionated plasma and may preserve information from otherwise labile signalling pathways. Their potential value does not depend on any single analyte, but on their ability to capture several pathobiological domains simultaneously.

**Figure 1:**
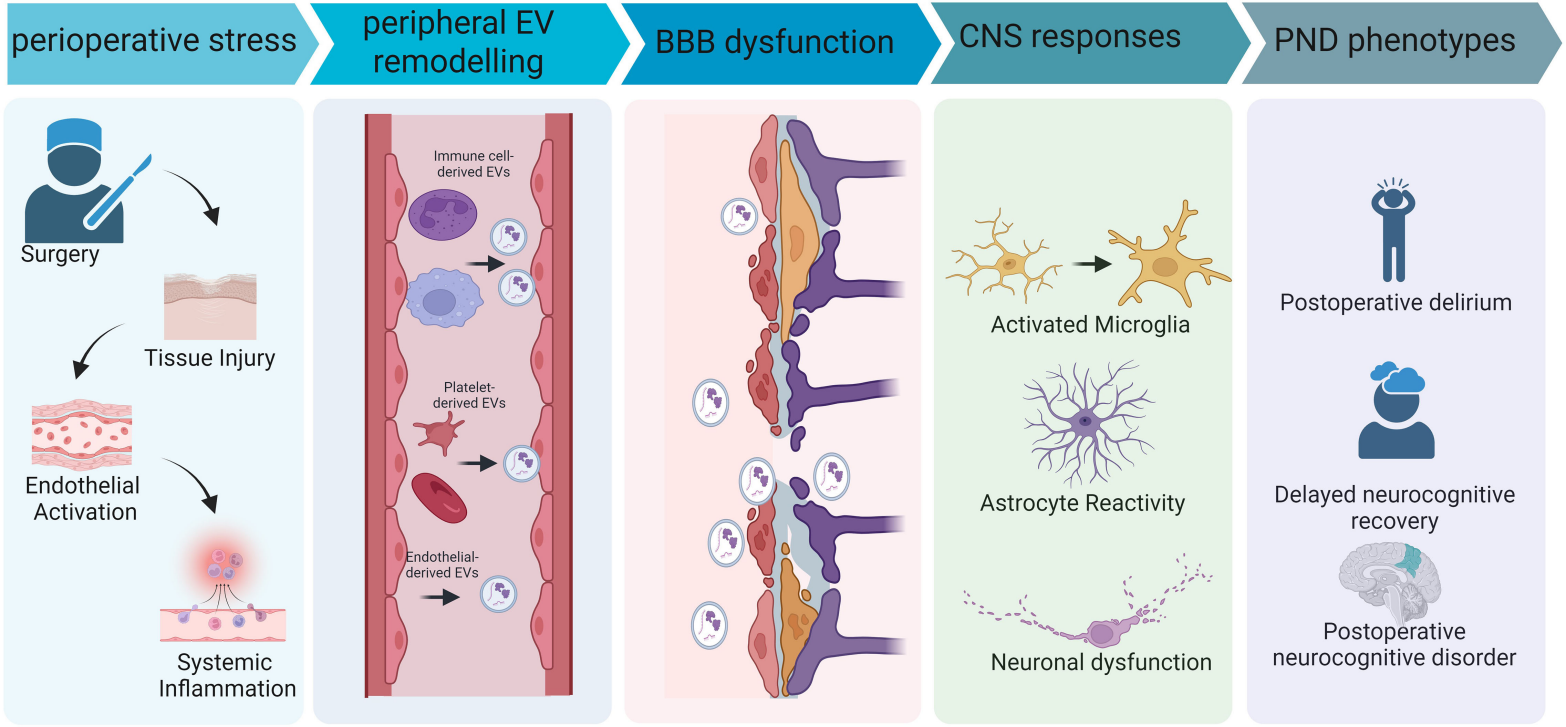
Proposed EV signalling framework in PND. The figure illustrates a proposed pathway from perioperative stress to PND phenotypes. Perioperative stress, including surgery-related tissue injury, endothelial activation, and systemic inflammation, may induce peripheral EV remodelling. Representative EV sources include immune cells, platelets, and endothelial cells. These EVs may interact with the blood–brain barrier and contribute to endothelial barrier disruption and exposure of CNS cells to circulating EV-associated signals. The BBB dysfunction panel depicts endothelial cells, perivascular cells, astrocytic endfeet, and circulating EVs at the vascular–brain interface. Subsequent CNS responses include microglial activation, astrocyte reactivity, and neuronal dysfunction, which may contribute to postoperative delirium, delayed neurocognitive recovery, and postoperative neurocognitive disorder. Arrows indicate the proposed direction of signalling rather than proven linear causality. BBB, blood–brain barrier; CNS, central nervous system; EVs, extracellular vesicles; PND, perioperative neurocognitive disorders.

Current clinical evidence supports this possibility, but remains exploratory. Studies in major orthopaedic surgery, cardiac surgery, and geriatric hip-fracture patients suggest that perioperative EV-associated cargo may help identify adverse neurocognitive outcomes, postoperative delirium, or delirium severity (10, 11, 39). These findings support the potential diagnostic and prognostic value of EV-associated signals because they connect circulating vesicle cargo with biological processes relevant to PND, including neuroinflammation, blood–brain barrier dysfunction, complement-related responses, and clinical symptom burden. However, these studies should be interpreted cautiously. EV preparations differ in biofluid source, sampling time, enrichment strategy, and downstream cargo analysis. Total plasma or serum EV preparations cannot fully resolve cellular source attribution, and perioperative factors such as coagulation activation, haemodilution, platelet release, lipoprotein interference, transfusion, and systemic inflammation may affect measured EV cargo. Moreover, most available studies are small, single-center, and associative, and no EV-based panel has yet been validated for routine diagnosis or prognosis. Evidence from other acute brain injury settings may also help refine this biomarker logic. For example, recent work in traumatic brain injury identified ceramide-rich EVs as pathogenic biomarker candidates, suggesting that lipid-defined EV subpopulations may carry clinically relevant neuroinjury information. Although this finding is not specific to PND, it supports the broader need to move beyond total EV counts toward cargo- and subtype-resolved EV biomarkers (41). At present, EV-associated markers should therefore be viewed as exploratory tools for biological stratification rather than established clinical biomarkers (**Table 1**, **Figure 1**).

**Table 1:** Representative clinical studies of EV-associated signals in PND.

Population / surgical setting	PND phenotype	EV material or cargo	Sampling time	Main finding
Older adults undergoing spine surgery (9)	Postoperative delirium	Plasma EV miRNAs	Preoperative	Preoperative EV miRNA profiles were associated with subsequent postoperative delirium.
Patients undergoing cardiac surgery (10)	Postoperative delirium	Exosomal protein/metabolite changes	Postoperative	Postoperative exosome-associated molecular changes were linked to delirium
Patients undergoing major orthopaedic surgery (11)	Poor postoperative neurocognitive outcome	EV miRNAs and C3	Serial postoperative sampling	Dynamic changes in plasma- and cerebrospinal fluid-derived EV cargo were associated with adverse neurocognitive outcome
Elderly patients undergoing spinal surgery (36)	Postoperative delirium	Bacterial EV taxa	Preoperative	Preoperative bacterial EV profiles showed predictive value for postoperative delirium
Geriatric patients undergoing hip fracture surgery (39)	Postoperative delirium	Exosomal α-synuclein	Perioperative	Perioperative exosomal α-synuclein increase was associated with delirium severity

## 5 Extracellular vesicle signalling in the pathobiology of PND

### 5.1 Perioperative stress and extracellular vesicle remodelling

The perioperative period provides a strong biological setting for EV release and cargo remodelling. Tissue injury, ischaemia–reperfusion, endothelial activation, coagulation, and systemic inflammatory responses can all increase circulating EV generation and reshape EV molecular composition (14, 37, 39, 42). In this context, the critical event is not simply an increase in vesicle number, but the emergence of EV populations enriched in inflammatory, vascular, metabolic, and tissue-injury signals. In a mouse orthopaedic surgery model, circulating EV proteins and miRNAs changed within hours after surgery and remained dynamically altered over the following days, indicating that EV remodelling is an early component of the response to surgical injury (37). Human studies discussed above are consistent with this concept, showing perioperative changes in EV cargo across orthopaedic, cardiac, and hip-fracture surgery settings (10, 11).

This early remodelling is relevant to PND pathogenesis because surgery-altered EVs may provide a circulating substrate through which systemic stress signals are transmitted to distant vascular, immune, and neural interfaces. This possibility is particularly plausible in procedures characterized by intense inflammatory, coagulation-related, and haemodynamic perturbation, such as major orthopaedic trauma and cardiopulmonary bypass (42). However, EV remodelling alone does not establish direct brain-directed causality. Rather, it defines an upstream perioperative event that may interact with soluble inflammatory mediators, endothelial dysfunction, and blood–brain barrier vulnerability to promote downstream CNS responses. In PND, perioperative stress should therefore be considered not only a trigger for soluble inflammatory mediators, but also a driver of EV remodelling that may contribute to later neurovascular and neuroinflammatory changes.

### 5.2 Extracellular vesicles at the systemic-to-brain interface

The pathogenic relevance of EVs in PND lies in their ability to convert peripheral injury into signals with neural consequences. Compared with freely soluble cytokines or damage-associated molecular patterns (DAMPs), EVs are more stable in circulation, protect labile cargo, and can deliver proteins, lipids, and nucleic acids to distant recipient cells (8, 43). This makes them plausible mediators of systemic-to-brain communication in a condition in which the initiating insult is extracranial but the phenotype is cerebral. In the broader literature on brain–periphery signalling, EVs are increasingly viewed as vehicles of inter-organ communication rather than passive cellular debris (44). The same concept is relevant to perioperative brain injury: the systemic response to surgery may be conveyed to the brain not only through soluble inflammatory mediators, but also through vesicle-associated molecular signals.

The strongest direct support for this view comes from transfer experiments. Gao et al. showed that circulating EVs isolated from aged mice after anaesthesia and surgery were sufficient to induce delirium-like behaviour in recipient aged mice (12). This finding indicates that postoperative EVs can carry biologically active information capable of altering brain function. At the same time, this does not require a simple model in which all pathogenic EVs cross an intact blood–brain barrier and directly enter the brain parenchyma. A more likely explanation is that systemic EVs act through more than one route: some may access central nervous system (CNS) cells under permissive conditions, whereas others may first act on endothelial or perivascular cells and thereby initiate secondary neural responses. This interpretation is more consistent with the biological complexity of perioperative brain injury.

### 5.3 Extracellular vesicle signalling in neurovascular dysfunction and neuroinflammation

Once perioperative EV signalling reaches the brain, the neurovascular unit is likely to be the first major site of interaction. In PND, blood–brain barrier dysfunction is increasingly regarded as a central pathogenic event rather than a late consequence of established brain injury (45). Clinical findings support this view. Postoperative increases in the cerebrospinal fluid-to-plasma albumin ratio have been associated with delirium and prolonged hospital stay in older adults after non-cardiac surgery (6), and a recent pilot study in cardiac surgery linked preoperative regional blood–brain barrier permeability to subsequent POD risk (46). These observations place the vascular–parenchymal interface near the centre of perioperative brain vulnerability.

Experimental studies outside the perioperative setting help clarify how EVs may contribute to this process. Cerebral microvascular endothelial cells release EV populations involved in blood–brain barrier regulation, and inflammatory conditions alter both their secretion profile and their biological effects (47). In a complementary study, cerebral endothelial cell-derived EVs crossed the blood–brain barrier, were taken up by neurons, astrocytes, and microglia, and showed enhanced microglial incorporation under inflammatory conditions (47). The same study showed that endothelial EV cargo, particularly miR-672-5p, could suppress TAB2/TAK1/NF-κB signalling and facilitate autophagic degradation of the NOD-like receptor family pyrin domain-containing 3 (NLRP3) inflammasome in microglia (48). Although these mechanisms have not been demonstrated directly in PND, they illustrate how EVs can act at the vascular–parenchymal interface and influence both barrier integrity and glial immune programming.

In this setting, EV signalling is well positioned to sustain neuroinflammatory responses. Neuroinflammation remains one of the best-supported mechanisms in PND, but cytokine release alone does not explain why cognitive dysfunction persists in only a subset of patients (13). EVs provide a plausible mechanism for maintaining and organizing inflammatory signalling within the CNS. In a postoperative cognitive dysfunction (POCD) model, M1-type microglia-derived EVs enriched in IL-1R1 aggravated cognitive dysfunction by promoting neuronal inflammation (13). Conversely, EVs derived from CCR5-modified microglia attenuated neuroinflammation and improved cognition, indicating that the biological effects of EVs depend on source state and cargo composition rather than on vesicle release alone (48). Together, these findings support a model in which perioperative EV signalling first perturbs the neurovascular interface and then contributes to a more sustained inflammatory circuit involving endothelial cells, microglia, and neurons.

### 5.4 From innate immune activation to synaptic and cognitive dysfunction

The downstream consequences of EV signalling in PND may extend beyond barrier injury and glial activation to include innate immune amplification, synaptic dysfunction, and behavioural impairment. One important link is complement-related signalling. Complement activation has been placed at the intersection of neuroinflammation and neurodegeneration in recent POD biomarker reviews (18), and a 2026 clinical study in elderly spine-surgery patients reported that perioperative complement profiling explained a substantial proportion of delirium severity variance (49). In the EV literature, the major orthopaedic surgery study by Mkrtchian et al. provides exploratory support for this connection, as EV-associated C3 was among the complement-related cargo changes associated with poor postoperative neurocognitive outcome (11). Although these findings do not establish that EV-associated complement directly causes synaptic injury, they suggest that complement-linked innate immune signalling may be part of the biological bridge between perioperative inflammation and postoperative brain dysfunction.

Synaptic dysfunction and neuronal injury may represent downstream consequences of this inflammatory and neurovascular cascade. Direct evidence in PND remains limited, but several experimental observations support the possibility that EVs can influence neuronal function. In aged rats, plasma exosomal miR-182-5p promoted neuroinflammation and cognitive dysfunction through BDNF suppression and NF-κB activation (38). In aged mice, circulating EVs collected after anaesthesia and surgery were sufficient to induce delirium-like behaviour in recipient animals (12). Outside the perioperative field, neuron-derived EVs have been shown to contain synaptic proteins, activate TrkB signalling, and regulate neuronal structural complexity (50). A recent cardiac surgery study further showed that patients who developed postoperative delirium had a greater perioperative increase in plasma sEV-cargo miR-330-3p, while *in vitro* overexpression of miR-330-3p increased tau phosphorylation and reduced neuronal viability. These findings suggest a potential connection between systemic EV-associated miRNA changes and tau-related neuronal injury (51). These findings suggest that EVs may affect neuronal and synaptic biology directly or indirectly, rather than acting only as peripheral inflammatory markers. In PND, the most plausible interpretation is a layered process in which neurovascular injury increases brain vulnerability, EV-associated innate immune signalling sustains local inflammation, complement-related pathways contribute to synaptic stress, and neuronal dysfunction progresses to delirium-like behaviour or longer-lasting cognitive decline. This sequence remains incompletely proven, but it provides a more coherent interpretation than a single-cause inflammatory model.

## 6 Source-specific extracellular vesicles in PND

Different EV populations are unlikely to contribute equally throughout the perioperative course. Their biological effects depend on the identity and activation state of the parent cell, and current evidence suggests that EVs derived from peripheral immune and vascular compartments are more relevant in the early postoperative phase, whereas CNS-derived EVs are more closely related to local inflammatory amplification and persistent neural dysfunction (4, 43, 52). Microbiota-associated EVs may represent an additional upstream source of biologically relevant signals. Although this sequence remains incomplete, it provides a more informative way to interpret the field than considering circulating EVs as a single, uniform population.

### 6.1 Peripheral immune and vascular extracellular vesicles

Peripheral immune and vascular EVs are likely to predominate in the early phase of perioperative signalling. Surgery rapidly activates circulating monocytes, macrophages, neutrophils, lymphocytes, endothelial cells, and platelets, all of which can release EVs carrying cytokines, lipids, enzymes, and regulatory RNAs relevant to inflammation, coagulation, endothelial activation, and vascular permeability (53–58). In this setting, immune cell-derived EVs are important less because they offer anatomical specificity than because they provide the first wave of biologically active circulating signals after perioperative stress. Recent experimental work supports this interpretation. In a two-hit inflammation model, macrophage-derived exosomes aggravated postoperative cognitive dysfunction by enhancing both peripheral and central inflammation, whereas pharmacologic inhibition of exosome release alleviated cognitive impairment (55). This finding gives peripheral immune EVs direct perioperative relevance rather than leaving them as a theoretical inflammatory source.

The vascular compartment is equally important. Endothelial- and platelet-derived EVs are especially relevant in major surgery because the perioperative state is accompanied by coagulation activation, haemodynamic instability, transfusion, and, in some patients, cardiopulmonary bypass (56). Platelet EVs are not simply by-products of haemostasis; they regulate leukocyte recruitment, endothelial permeability, and inflammatory signalling (57, 58). Endothelial EVs, meanwhile, are positioned at the blood–brain interface and therefore provide a plausible link between systemic surgical stress and neurovascular injury. Even when brain-derived EVs remain difficult to identify in blood, peripheral immune and vascular EVs are likely to constitute the major circulating compartment through which early systemic injury signals are delivered.

### 6.2 Central nervous system-derived extracellular vesicles

Once the CNS is engaged, source-specific EVs derived from microglia, astrocytes, and neurons are likely to shape the local trajectory of injury. Among these, microglia-derived EVs currently have the strongest direct mechanistic link to PND. Microglia are central to postoperative neuroinflammation, and once activated they do not act solely through soluble cytokines; they also release EVs that redistribute inflammatory signals to nearby neurons and glia (59). In a POCD model, M1-type microglia-derived EVs enriched in IL-1R1 aggravated cognitive dysfunction by promoting neuronal inflammation (13). A subsequent study showed the opposite pattern: EVs derived from CCR5-modified microglia attenuated neuroinflammation and improved cognition (48). These findings indicate that microglial EVs are not intrinsically harmful; their effects depend on activation state and cargo composition. In PND, their importance lies mainly in sustaining and propagating local inflammatory signalling after the initial systemic insult.

Astrocyte- and neuron-derived EVs are likely to become more important as the field moves beyond broad descriptions of inflammation toward cell-type-specific readouts of injury and recovery. Astrocyte-derived EVs have been implicated in neuroimmune modulation, blood–brain barrier support, and communication between the CNS and the periphery, and may either propagate injury or support restoration depending on context (60). Neuron-derived EVs are particularly relevant to persistent cognitive dysfunction because they are closer to synaptic and neuronal stress. Experimental studies have shown that neuron-derived EVs contain synaptic proteins, promote spine formation, activate TrkB signalling, and preserve neuronal complexity (50). From a translational perspective, CNS-derived EVs are also attractive because they may improve the anatomical specificity of blood-based biomarker studies, although enrichment strategies and marker specificity remain unresolved (20, 61). In PND, direct studies on astrocyte- and neuron-derived EVs remain limited, but this source category is likely to be important for understanding why some patients show mainly inflammatory phenotypes whereas others develop more persistent synaptic and cognitive dysfunction.

### 6.3 Microbiota-associated extracellular vesicles

Gut microbiota-associated EVs represent the most exploratory but potentially important source category in PND. They are relevant because perioperative stress can disrupt gut barrier integrity, alter microbial composition, and modify host–microbe signalling, particularly in older patients and after major surgery. Microbiota-derived EVs can enter the circulation, carry bacterial or metabolically active cargo, and influence systemic immunity, barrier function, and neuroinflammatory pathways (8, 62). In neurological disease more broadly, they are increasingly discussed as effectors of gut–brain communication rather than indirect correlates of dysbiosis (8). In PND, the most direct human evidence comes from a prospective spinal-surgery cohort in which preoperative circulating bacterial EV profiles showed predictive value for postoperative delirium (36). This does not establish causality, but it suggests that microbiota-associated vesicles may capture a dimension of host vulnerability that is not adequately reflected by conventional plasma biomarkers. At present, this source category is less mechanistically defined than microglial or vascular EVs, but it broadens the PND framework beyond sterile surgical injury and points to a potential role for perioperative dysbiosis, barrier dysfunction, and remote immune signalling.

## 7 Therapeutic implications of extracellular vesicle signalling

Current therapeutic strategies related to EV signalling in PND can be considered from two directions: limiting pathogenic EV activity and exploiting beneficial EVs as therapeutic tools (**Table 2**). The first direction is supported by perioperative experimental studies. In a POCD model, EVs released from activated microglia aggravated neuroinflammation, synaptic injury, and cognitive impairment, whereas CCR5-modified microglial EVs attenuated these changes, indicating that pathogenic EV signalling is not static and may be therapeutically redirected (48). In parallel, antler mesenchymal stem cell-derived exosomes improved cognition and reduced hippocampal injury in cardiopulmonary bypass rats, at least in part through inhibition of the TLR2/TLR4-MyD88-NF-κB pathway (63). Although these studies remain preclinical, they suggest that EVs are not only markers of perioperative brain injury but also modifiable disease effectors. A related observation comes from exosome-centered multi-omics in postoperative delirium, where Connectivity Map analysis identified candidate small molecules targeting exosome-associated inflammatory pathways, including MMP9-related signalling (10). Taken together, these findings support the view that perioperative neuroinflammation may be influenced not only by soluble mediators but also by interventions directed at pathogenic EV generation, cargo, or downstream signalling.

**Table 2:** Current therapeutic directions of extracellular vesicle signalling in PND.

Strategy	Representative evidence	Therapeutic rationale	Key limitation
Suppressing pathogenic EV signalling	CCR5-modified microglial EVs reduced neuroinflammation and improved cognition in a POCD model (48)	Redirecting EV-driven inflammatory signalling	Evidence limited to preclinical models
Stem cell-derived protective EVs	Antler mesenchymal stem cell-derived exosomes improved cognition in cardiopulmonary bypass rats (63)	Neuroprotection via inhibition of TLR2/TLR4-MyD88-NF-κB signalling	Product heterogeneity and unclear clinical translation
Microbiota-derived protective EVs	*Akkermansia muciniphila*-derived EVs improved cognition, preserved barrier integrity, and reduced inflammation in POCD models (64, 65)	Modulation of gut-brain communication and inflammasome activity	Mechanistic specificity and reproducibility remain limited
Anti-inflammatory microbial EVs	Lactobacillus-derived EVs reduced pro-inflammatory cytokines and promoted an M2-like microglial phenotype *in vitro (*66)	Reprogramming microglial inflammatory responses	No direct *in vivo* PND validation
Targeting EV-related pathways	Exosome-centered multi-omics identified candidate compounds targeting exosome-associated inflammatory pathways (10)	Indirect modulation of pathogenic EV signalling	Exploratory only; no therapeutic validation
Engineered EVs for CNS delivery	Broader EV literature supports engineered EVs for brain-directed drug/RNA delivery (67–69)	Delivery platform for perioperative neuroprotection	Most evidence comes from non-PND models

A second line of investigation focuses on naturally protective or engineered EVs. Here, direct evidence in PND is still limited but expanding. Beyond stem cell-derived vesicles, microbiota-associated EVs have entered the field. Small extracellular vesicles derived from Akkermansia muciniphila improved cognitive function, preserved intestinal barrier and blood-brain barrier integrity, and reduced inflammation and microglial activation in POCD mice (64). A later study extended this observation by showing that Akkermansia muciniphila-derived EVs promoted cognitive recovery in aged mice under sevoflurane anaesthesia and inhibited NLRP3 inflammasome activation (65). Lactobacillus-derived EVs have also shown anti-inflammatory effects in an *in vitro* PND-relevant microglial model, where they reduced IL-1β and IL-6, increased IL-10, and shifted microglia toward an M2-like state (66). These findings do not yet define a mature therapeutic strategy, but they broaden the field beyond mesenchymal stem cell EVs and suggest that beneficial vesicles may act through several mechanisms, including suppression of innate immune signalling, preservation of barrier integrity, and modulation of gut–brain communication.

The broader EV therapeutics literature is also relevant because it defines what may become feasible for PND before disorder-specific clinical studies are available. Recent reviews emphasize that EVs are attractive CNS delivery vehicles because of their biocompatibility, modifiability, and capacity to cross or bypass brain barriers, particularly when combined with surface engineering, cargo optimization, or brain-targeting ligands (67, 68). Intranasal delivery has attracted particular interest because it may reduce systemic exposure and partially bypass the blood-brain barrier, while engineered EVs are increasingly being developed as carriers for drugs, RNAs, and other biological payloads (69, 70). For PND, this suggests a plausible translational direction: EVs may eventually be used as programmable delivery systems to suppress perioperative neuroinflammation, stabilize the neurovascular interface, or protect synaptic function during the vulnerable postoperative period. At present, however, most of the supporting work comes from broader CNS or inflammatory disease models rather than from PND itself.

From a clinical perspective, EV-based therapeutic strategies should be considered according to patient risk, timing, and disease stage rather than as a uniform intervention. In high-risk patients, including older or frail individuals, patients with pre-existing cognitive impairment, and those undergoing cardiac, major orthopaedic, or spine surgery, EV-based biomarkers may first be useful for identifying biological vulnerability before clinical symptoms emerge. Preventive or very early postoperative approaches would therefore be most relevant during this stage, particularly interventions aimed at reducing pathogenic EV release or modulating gut–brain and inflammatory signalling. During the acute postoperative phase, when systemic inflammation, endothelial activation, and blood–brain barrier dysfunction are prominent, experimental strategies that attenuate pathogenic immune or microglial EV signalling may be more appropriate. In later stages, including delayed neurocognitive recovery and postoperative neurocognitive disorder, therapeutic or engineered EVs may be conceptually more suitable for targeting persistent neuroinflammation, synaptic stress, or neuronal repair. However, this stage-based allocation remains hypothetical because direct clinical evidence in PND is not yet available.

The main barriers to translation remain substantial. Large-scale production is still difficult, cargo loading is variable, and targeting efficiency is often insufficient for reliable brain delivery. Standardized dose-response frameworks, potency assays, and harmonized clinical protocols are still lacking even for mesenchymal stromal cell-derived EV platforms, and recent reviews continue to identify dosing and manufacturing consistency as major unmet needs (71, 72). Route of administration is another unresolved issue: systemic delivery is limited by rapid clearance and off-target sequestration, whereas intranasal delivery, although attractive for CNS applications, still faces problems with reproducibility and tissue penetration (68). These concerns are longstanding. The ISEV position paper on clinical translation made clear that EV-based therapeutics require rigorous control of source material, manufacturing, product characterization, safety, and regulatory compliance before clinical use can be justified (73). For PND, the immediate value of this field therefore lies less in near-term clinical application than in identifying which pathogenic EV signals should be blocked, which protective vesicle populations are worth developing, and which delivery strategies are realistic for perioperative brain protection.

## 8 Challenges and future directions

Despite growing interest in EV signalling in PND, the field remains at an early stage. One major challenge is phenotypic heterogeneity. Postoperative delirium, delayed neurocognitive recovery, and more persistent postoperative neurocognitive disorder differ in timing, clinical context, and likely biology, yet many EV studies still discuss them together or rely on experimental “POCD” models that do not fully align with current perioperative nomenclature (3, 4). Human data are also limited by small sample sizes, single-center designs, and narrow surgical populations, with most direct evidence derived from orthopaedic or spine surgery cohorts (11, 14). At this stage, the priority is therefore not simply to identify additional candidate cargos, but to establish prospective studies with clearer phenotypic definitions, standardized cognitive endpoints, and serial perioperative sampling.

A second challenge is source attribution. Total plasma EVs represent a biologically mixed population, and current methods still do not reliably distinguish vesicles derived from peripheral immune and vascular compartments from those that reflect CNS signalling (74, 75). This issue is particularly important in PND, where the key question is not whether circulating EVs change after surgery, but which EV populations are actually relevant to perioperative brain injury. Standardization is therefore essential. Both MISEV2023 and MIBlood-EV emphasize that EV data are highly sensitive to sample type, processing delay, centrifugation strategy, storage, and downstream characterization (29). In perioperative studies, these variables become even more difficult to control because inflammation, coagulation, haemodilution, and transfusion can all alter the measurable vesicle pool.

The third challenge is causality. Much of the current literature remains associative rather than mechanistic. Future work will require source-resolved transfer experiments, cell-specific perturbation studies, matched blood–cerebrospinal fluid analyses, and study designs that distinguish early systemic EV signalling from later CNS-derived amplification. Translation also remains limited by unresolved issues in manufacturing, potency testing, dose definition, biodistribution, and regulatory oversight (76, 77). For PND, the most realistic near-term goal is not immediate clinical application, but a clearer translational pathway: identify which EV populations matter, define when they matter, determine whether they drive or merely reflect injury, and then connect those findings to standardized assays and mechanism-based interventions. If that sequence can be established, EV research may help explain why a systemic surgical insult leads to persistent brain dysfunction in only a subset of patients.

## 9 Conclusions

Extracellular vesicle signalling provides a plausible framework for understanding how systemic perioperative stress is translated into postoperative brain dysfunction. Current evidence suggests that EVs may participate in the progression from peripheral inflammation and vascular stress to blood–brain barrier dysfunction, neuroinflammation, synaptic injury, and cognitive decline. EV-associated signals also show promise as biomarker candidates and, in preclinical studies, as potential therapeutic targets. At present, however, the field remains constrained by heterogeneous clinical phenotypes, limited human data, incomplete source attribution, and methodological variability. Future progress will depend on phenotype-defined prospective studies, standardized EV workflows, and source-resolved mechanistic investigation. With these advances, EV research may offer both a clearer understanding of PND pathogenesis and a stronger basis for biomarker development and perioperative neuroprotection.
